# Divergent CPEB prion-like domains reveal different assembly mechanisms for a generic amyloid-like fold

**DOI:** 10.1186/s12915-021-00967-9

**Published:** 2021-03-11

**Authors:** Rubén Hervás, María del Carmen Fernández-Ramírez, Albert Galera-Prat, Mari Suzuki, Yoshitaka Nagai, Marta Bruix, Margarita Menéndez, Douglas V. Laurents, Mariano Carrión-Vázquez

**Affiliations:** 1 Instituto Cajal, IC-CSIC, Avda. Doctor Arce 37, E-28002 Madrid, Spain; 2 Present address: School of Biomedical Sciences, Li Ka Shing Faculty of Medicine, The University of Hong Kong, Pokfulam, Hong Kong SAR, China; 3 Department of Degenerative Neurological Diseases, National Institute of Neuroscience, National Center of Neurology and Psychiatry, Kodaira, Tokyo Japan; 4 Present address: Diabetic Neuropathy Project, Department of Sensory and Motor Systems, Tokyo Metropolitan Institute of Medical Science, Setagaya, Tokyo Japan; 5 Present address: Department of Neurology, Faculty of Medicine, Kindai University, Osaka-Sayama, Osaka, Japan; 6 Instituto de Química-Física Rocasolano, IQFR-CSIC, Serrano 119, E-28006 Madrid, Spain; 7 Centro de Investigación Biomédica en Red sobre Enfermedades Respiratorias (CIBERES), C/ Monforte de Lemos 3-5, 28029 Madrid, Spain

**Keywords:** Cytoplasmic polyadenylation element binding protein (CPEB), Functional amyloids, Prion-like protein, Memory persistence, Coiled coil

## Abstract

**Background:**

Amyloids are ordered, insoluble protein aggregates, characterized by a cross-β sheet quaternary structure in which molecules in a β-strand conformation are stacked along the filament axis via intermolecular interactions. While amyloids are typically associated with pathological conditions, functional amyloids have also been identified and are present in a wide variety of organisms ranging from bacteria to humans. The cytoplasmic polyadenylation element-binding (CPEB) prion-like protein is an mRNA-binding translation regulator, whose neuronal isoforms undergo activity-dependent aggregation, a process that has emerged as a plausible biochemical substrate for memory maintenance. CPEB aggregation is driven by prion-like domains (PLD) that are divergent in sequence across species, and it remains unknown whether such divergent PLDs follow a similar aggregating assembly pathway. Here, we describe the amyloid-like features of the neuronal *Aplysia* CPEB (ApCPEB) PLD and compare them to those of the *Drosophila* ortholog, Orb2 PLD.

**Results:**

Using in vitro single-molecule and bulk biophysical methods, we find transient oligomers and mature amyloid-like filaments that suggest similarities in the late stages of the assembly pathway for both ApCPEB and Orb2 PLDs. However, while prior to aggregation the Orb2 PLD monomer remains mainly as a random coil in solution, ApCPEB PLD adopts a diversity of conformations comprising α-helical structures that evolve to coiled-coil species, indicating structural differences at the beginning of their amyloid assembly pathways.

**Conclusion:**

Our results indicate that divergent PLDs of CPEB proteins from different species retain the ability to form a generic amyloid-like fold through different assembly mechanisms.

**Supplementary Information:**

The online version contains supplementary material available at 10.1186/s12915-021-00967-9.

## Background

New biological traits can emerge from heritable changes in protein-based epigenetic elements known as prions [1–5], redefining the central dogma in which the heritable information is stored in nucleic acids [6]. A prion-like protein family with a defined role in memory persistence in different species, from *Aplysia* to mammals, is the mRNA-binding CPEB family. Discovered first in oocytes as regulators of mRNA translation [7], the aggregated state of neuronal-specific isoforms of CPEB family members was later found to play a key role in (i) long-term synaptic facilitation in *Aplysia* [8], (ii) long-term potentiation of synaptic transmission in mice [9], and (iii) maintenance of long-term memory in both mice and *Drosophila* [9–16], through the activation of dormant mRNAs mediated by the acquisition of a self-sustaining amyloid state, at least in *Drosophila* [17].

**Fig. 1:**
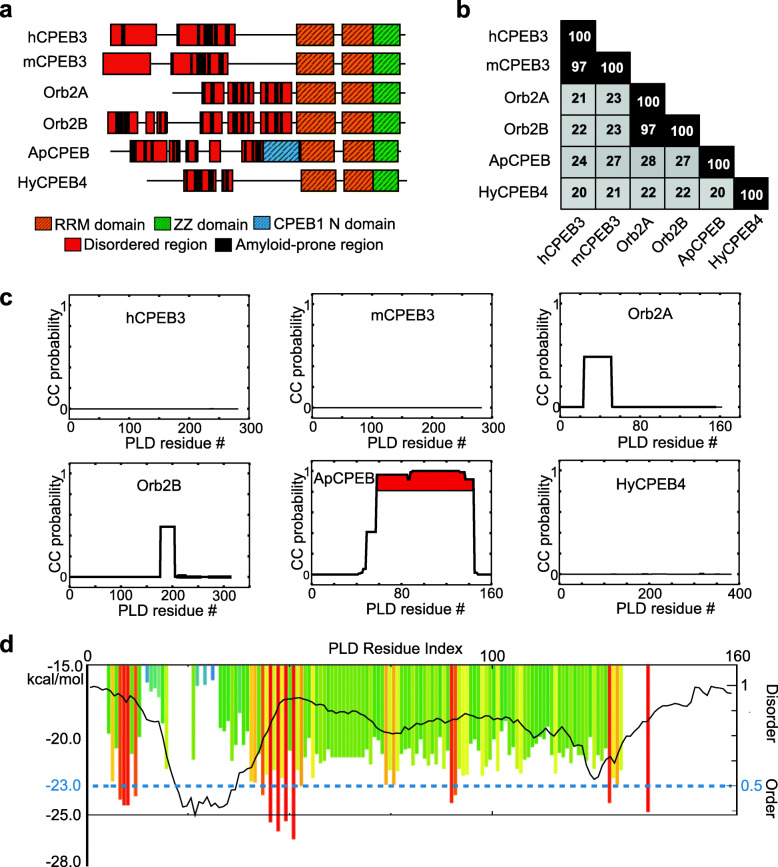
Computational sequence analysis of neuronal CPEB orthologs. **a** Domain analysis and organization. The selected CPEB proteins have a defined role in memory persistence in different species. CPEB4 from *Hydra magnipapillata*, a fresh-water member of the cnidarian, was selected as the most primitive animal to have neurons. The two RRMs (orange) and ZZ domain (green) are located in the conserved C-termini. The divergent N-termini of all analyzed CPEB proteins, where the PLD is located, show amyloid-prone (black boxes) and disordered (red boxes) segments. **b** Pairwise sequence identity of the N-terminal region represented as a matrix, from the first residue to the first residue of RRM1. ApCPEB PLD and Orb2A PLD share 28% sequence identity. **c** CC probability (from 0 to 1, per residue) obtained using the Coils algorithm for N-terminal regions of neuronal CPEB isoforms. ApCPEB PLD is the only domain with segments whose CC formation propensity is over 0.8 (highlighted in red), followed by *Drosophila* Orb2A PLD and Orb2B PLD with a propensity near 0.5. **d** Prediction of disorder propensity using PONDR-FIT, and amyloid-spine formation using ZipperDB, along the 160 residues of the ApCPEB PLD sequence. The dark line shows the predicted disorder score per residue. Values above or below 0.5 predict disordered or ordered, respectively. Each colored bar indicates the hexapeptide starting position for amyloid-spine prediction. Segments with lower energy than the threshold at -23 kcal/mol are indicated in red as prone-amyloid segments

Neuronal-specific isoforms of CPEB family members confer a similar structure, containing a C-terminal RNA binding domain and an N-terminal PLD. Sequence analysis revealed that the amino acid sequence of the folded C-terminus of neuronal CPEB proteins, which comprises two RNA-recognition motifs (RRM) and a ZZ-type zinc finger domain necessary for efficient mRNA binding [18], is conserved across species (Fig. 1a, Additional file 1: Figure S1a, b). By contrast, the flexible N-terminus, which comprises the PLD responsible for the formation of sodium dodecyl sulfate (SDS)-resistant aggregates of ApCPEB, Orb2, and CPEB3 in the adult brain of *Aplysia*, *Drosophila*, and vertebrates, respectively [10, 17, 19, 20], is divergent across species (Fig. 1a, b, Additional file 1: Figure S1c). In the *Drosophila* nervous system, Orb2 exists in diverse conformational states, from a monomeric state to a self-sustaining amyloid state [17]. While Orb2 monomers repress synaptic translation, the amyloid state enhances synaptic translation [11, 17]. Similarly, the neuronal-specific ApCPEB isoform can exist in at least two different conformational states: a soluble form and a β-sheet-rich amyloid-like form, which has an enhanced binding capacity to target mRNAs [20, 21]. In both ApCPEB and Orb2 proteins, in spite of their low sequence identity (Fig. 1a, b, Additional file 1: Figure S1c), the N-terminal PLD plays a key role in the transition to a β-sheet-rich fold through a neuronal activity-dependent aggregation process [10, 17, 19, 20]. However, the commonalities and/or differences in the conformational transition that trigger the assembly of such divergent PLDs remain unknown. Here, to get insight into this question, the assembly pathway of the neuronal *Aplysia* CPEB PLD (residues 1–160) is structurally characterized from the monomeric to the aggregated state. Upon comparison with that of the *Drosophila* Orb2A PLD (residues 1–162) [16], their divergent PLDs were found to aggregate through alternative amyloid assembly pathways. Since functional protein aggregation must be physiologically regulated, the selection through evolution of divergent PLD sequences in members of the same protein family might have conferred a specific feature. The existence of alternative amyloid assembly pathways, as here described, could attend to the potential, diverse regulatory mechanisms in each organism.

## Results

The role of coiled coils (CCs) in aggregation and activity of glutamine-asparagine (Q/N)-rich proteins in vivo has been previously reported [22]. The neuronal-specific isoforms of CPEB family members have Q/N-rich N-termini that resemble a yeast PLD [23]. In search of shared features among CPEB family members, the predicted CC propensity of selected CPEB PLDs from different species was examined. By using the Coils algorithm [24], ApCPEB PLD (ApCPEB_1–160_) scored as the highest predicted hit to contain CC domains (Fig. 1c). Besides the inherent tendency to form intermolecular CCs, the ApCPEB PLD sequence contains regions prone to be disordered and form amyloid spines (Fig. 1d). In agreement with Coils, ApCPEB PLD was predicted to contain CC domains, which overlapped with the poly-Q stretches, by using three additional algorithms that detect CC motifs in protein sequence: PairCoil2 [25], Marcoil [26], or PCOILS [27] (Additional file 1: Figure S2). The next hit, with a lower predicted probability to form CCs, corresponded to the PLD from *Drosophila* Orb2A (Orb2A_1–161_) and Orb2B (Orb2B_1–373_), which were previously reported to form mainly a random coil conformation in solution by Circular Dichroism (CD) [16]. The predicted CC propensity of the mouse, human, and hydra orthologs N-termini (mCPEB3_1–283_, hCPEB3_1–282_ and hyCPEB4_1–383_, respectively) was insignificant (Fig. 1c).

Compatible with the CC computational prediction, ApCPEB PLD showed a dominant α-helical CD spectrum (Additional file 1: Figure S3a), similar to the reported for the full-length ApCPEB [21]. After 72-h incubation, the CD signal decreased to approximately 10% of the initial value (Additional file 1: Figure S3a). The Q-binding peptide 1 (QBP1), a peptide known to block the β-structure transition in the monomer and the subsequent aggregation of poly-Q containing proteins [16, 28–30], as well as a scrambled version of it (SCR, [31]), were used to determine the nature of the CD signal loss. ApCPEB PLD physically interacted with QBP1 through an exothermic reaction, as revealed by isothermal titration calorimetry (ITC) (Additional file 1: Figure S3b). By contrast, according to the net heat exchange, SCR did not show a significant interaction (Additional file 1: Figure S3b). These data suggest that the reported loss of CD signal is likely due to ApCPEB PLD aggregation over time mediated by the poly-Q stretches and that QBP1 was able to lower it (Additional file 1: Figure S3a).

**Fig. 2:**
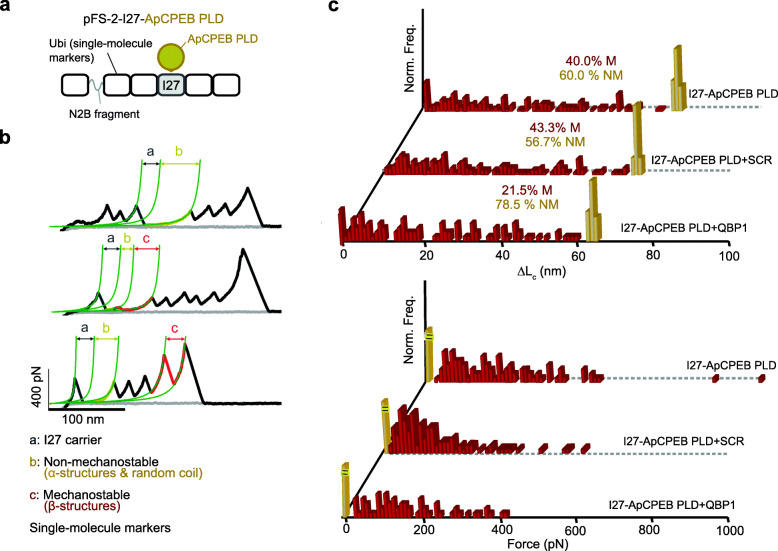
AMF-SMFS characterization of the ApCPEB PLD monomer. **a** Schematic cartoon of pFS-2 vector using I27 as a carrier (gray), hosting ApCPEB PLD (yellow) as a guest. Ubi molecules used as single-molecule markers are represented in black. The polyprotein encoded by pFS-2 contains a random coil region (a fragment of titin, N2B, gray line), acting as a spacer to overcome the noisy proximal region of the force-extension recordings. **b** Representative force-extensions recordings. Traces in yellow correspond to NM events. M events, in red, show different mechanical stability (*F*_*u*_) and increased contour length (Δ*L*_*c*_) values. “b”+“c” values complete the length of the fully stretched ApCPEB PLD. **c** Δ*L*_*c*_ and *F*_*u*_ histograms for polyproteins carrying ApCPEB PLD reveal a rich conformational polymorphism in terms of Δ*L*_*c*_ (top panel) and *F*_*u*_ (bottom panel), ranging from NM events (yellow bars) to M events (red bars, *n* = 145). In the presence of QBP1, the mechanical conformational polymorphism is lowered, while SCR has no apparent effect (QBP1; *n* = 186, SCR; *n* = 144)

Next, to analyze the conformational space of monomeric ApCPEB PLD prior aggregation, single-molecule force spectroscopy based on atomic force microscopy (AFM-SMFS) was used in the length-clamp mode. Together with a protein engineering strategy, AFM-SMFS was previously used to analyze the conformational diversity of amyloid-forming proteins at the monomer level [16, 30, 32, 33]. In this strategy, known as the “carrier-guest” strategy, the ApCPEB PLD is mechanically protected inside a carrier protein whose coding region is present in the plasmid used for these force spectroscopy studies (pFS-2) [34] (Fig. 2a). The I27 module from human cardiac titin, or alternatively the protein Ubiquitin (Ubi), were used as carrier proteins since both display known mechanical properties [35, 36] (Additional file 1: Figure S4a). Using this strategy, the ApCPEB PLD single-molecule data are recorded after the unfolding of the carrier, far from the proximal region to the AFM substrate, known to be noisy and that therefore typically generates unreliable signals [34]. Prior to the AFM-SMFS analysis, nuclear magnetic resonance (NMR) spectroscopy was used to determine the structural integrity of the I27 carrier upon ApCPEB PLD insertion. The comparison of 2D ^1^H nuclear Overhauser effect spectroscopy (NOESY) data from the isolated ApCPEB PLD versus the ApCPEB PLD nested into the I27 fold showed only slight shifts in the variant bearing ApCPEB PLD (Additional file 1: Figure S5). These data reveal that the I27 carrier retains its native, folded structure upon ApCPEB PLD insertion, evincing the reliability of the mechanical protection strategy used for the AFM-SMFS nanomechanical measurements.

Using the length-clamp mode of AFM-SMFS, two basic parameters are directly accessed: (i) the mechanical stability of the resistance barriers (unfolding force, *F*_*u*_), measured by the height of the force peaks in a force-extension recording, and (ii) the contour length released after unfolding (increase in contour length, Δ*L*_c_), measured by fitting the force-extension recording to the worm-like chain (WLC) model of polymer elasticity [37]. The AFM-SMFS analysis showed that the monomeric ApCPEB PLD fluctuates over a conformational space that includes two major populations: 60.0% of non-mechanically resistant conformers (NM; previously defined as *F*_*u*_*,* ≤ 20 pN) and 40.0% of mechanically resistant conformers (M; *F*_*u*_> 20 pN) (Fig. 2b, c and Additional file 1: Figure S4b-e). The M conformers spanned a range of mechanical stabilities or resistance barriers (*F*_*u*_) located at different places of the molecule, which are measured by the length released upon unfolding (Δ*L*_*c*_) (Fig. 2b, c, Additional file 1: Figure S4c-e, and Supplementary Table 1). The lack of correlation between *F*_*u*_ and Δ*L*_c_ indicates that no specific regions mediate the mechanical stability in the M conformers (Fig. 2c, Additional file 1: Figure S6a and Supplementary Table 1), a population that often contains more than one region of mechanical resistance per molecule (Additional file 1: Figure S4d-e and Supplementary Table 1). To rule out artifactual effects derived from spurious interactions due to the use of a specific carrier (I27), Ubi was employed as an alternative carrier [34, 35], obtaining a similar distribution: 57.9% NM and 42.1% M conformers (Additional file 1: Figure S6b,c). M recordings are mainly attributed to the unraveling of the β-structures [38], although low forces, still detected by AFM, could be due to the mechanical unfolding of intramolecular CCs [39–42]. By contrast, NM events could originate either from the stretching of random coil or isolated α-helix conformers [38]. The latter might contribute to the formation of intermolecular CCs at a later stage in the ApCPEB PLD assembly, as computationally predicted (Fig. 1c and Additional file 1: Figure S2), and similarly to other Q/N-rich peptides that form dimers and higher-order multimeric CCs [22]. QBP1, which prevents the β-sheet conformational transition of the α-helical poly-Q monomer [28], lowered the formation of M conformers (Fig. 2c and Additional file 1: Figure S6d). However, neither SCR nor DMSO, used as QBP1/SRC vehicle, had a discernible effect (Fig. 2c and Additional file 1: Figure S6d,e), suggesting that the ApCPEB PLD assembly pathway is initially driven by the poly-Q stretches. Considering the CC prediction, the CD, and AFM-SMFS data together, it can be concluded that the conformational diversity of ApCPEB PLD is similar to that of the Orb2 PLD at the monomer level [16], but likely with larger populations of α-helical structures.

**Fig. 3:**
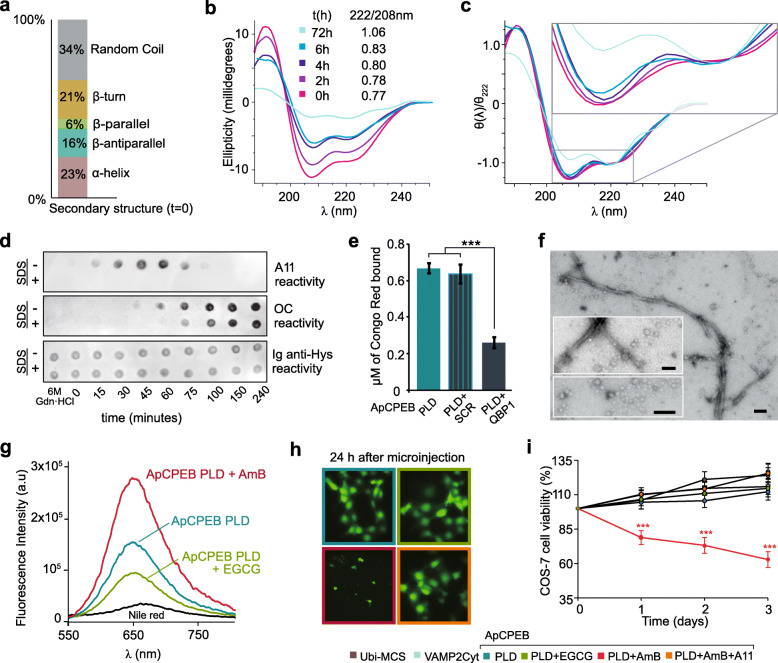
Dissection of ApCPEB PLD amyloid-like assembly pathway. **a** Secondary structure content of soluble ApCPEB PLD conformers at 0h derived from the far-UV CD spectrum deconvolution. **b** Raw far-UV CD spectra of ApCPEB PLD over time. At zero time, the spectrum shows minima at 222 and 208 nm and a maximum at around 195 nm, characteristic of the α-helical structure. A loss of signal with time is accompanying an increase in the θ_222/208nm_, which is associated with CCs formation. **c** Concentration-independent CD spectra normalized by the ellipticity signal value at 222 nm. The change of shape with time can be better appreciated when the relative intensities of the spectra, with relation to the respective value at 222 nm, are compared to compensate for the loss of signal. Normalized spectra do not overlap, particularly at 72 h, showing the occurrence of structural changes over time. **d** Dot blot analysis of on-pathway ApCPEB multimers formed during the assembly. SDS-sensitive A11-reactive oligomeric species appear from ~ 15 to 75 h and evolve to SDS-resistant OC-reactive, amyloid-like aggregates beyond that time point. **e** Concentration (μM) of the azo-dye CR bound to ApCPEB PLD amyloid-like species. ApCPEB PLD amyloid-like formation is lowered in the presence of QBP1. Data are expressed as mean ± SEM: ***p< 0.001 (One-way ANOVA and Tukey post-test). **f** Representative electron micrographs of ApCPEB PLD oligomers and amyloid-like filaments. Scale bar: 50 nm. **g** Nile Red fluorescence emission spectra of ApCPEB PLD, ApCPEB PLD:AmB, and ApCPEB PLD:EGCG complexes. While treatment with AmB increases the Nile red fluorescence intensity, EGCG causes the opposite effect. **h** Fluorescence micrographs of COS-7 cells 24 h after the microinjection of indicated samples along with fluorescein-labeled dextran. ApCPEB PLD:AmB microinjection decreases the number of live cells after 24 h. Scale bars: 100 μm. **i** Survival curves of COS-7 microinjected cells. None of the samples but the ApCPEB PLD species trapped with AmB cause toxicity. The toxic phenotype was rescued by the A11 antibody. Data are expressed as the mean ± SEM: ***p < 0.00 (red asterisks) ApCPEB PLD:AmB versus remaining samples; two-way ANOVA and Bonferroni post-test. Ubi-MCS and the cytoplasmic region of VAMP2 are used as controls of a folded and disordered protein, respectively. No. of cells microinjected per sample: *n* = 100–200 cells, 3 replicates

Next, the primary structural changes associated with protein assembly, such as the formation of intermolecular CCs, were monitored by far-UV CD. The ApCPEB PLD CD spectrum exhibited two negative dichroic minima at 222 nm and 208 nm and a positive dichroic band with a maximum at around 195 nm, characteristic of proteins with high α-helix content (Additional file 1: Figure S3a). CD spectra deconvolution using a reference dataset revealed a large proportion of structured conformations acquired by ApCPEB PLD (Fig. 3a). By contrast, its ortholog Orb2A PLD remains as a random coil in solution, as characterized by CD spectroscopy [16], adopting minor populations of α-helix in the Q/H-rich region at neutral pH, as detected by NMR spectroscopy [43]. At time zero, the far-UV CD spectra of ApCPEB PLD revealed a ratio between the 222 nm and 208 nm ellipticity signals (θ_222/208nm_) lower than 0.8 (Fig. 3b), which is taken as indicative of the presence of isolated α−helices [44–46]. However, sample aging produces a gradual loss of signal due to protein aggregation that is accompanied by an increase in the θ_222/208nm_ ratio. In particular, the spectrum reached a θ_222/208nm_ = 1.06 at 72 h, which is associated with the formation of CCs (Fig. 3b) [44–46]. Concentration-independent CD spectra at 72 h, normalized by the signal value at 222 nm, differ in shape from those obtained at short incubation times, suggesting that the observed differences are due to structural rearrangements over time (Fig. 3c). Additional evidence of a CC-based aggregation mechanism was obtained by analyzing a 20-mer peptide, whose sequence (E_121_QLQQQQLQLQQQLQQQLQH_140_) corresponds to part of the C-terminal region of the ApCPEB PLD (Additional file 1: Figure S7). NMR and fluorescence resonance energy transfer (FRET) measurements suggest an anti-parallel CC as the most stable configuration adopted for ApCPEB PLD_121–140_, which can be destabilized by disrupting the hydrophobic interface between two alpha helices via three Leu to Lys substitutions (Additional file 1: Figure S7). Taken together, these observations imply the formation of CCs at the early ApCPEB PLD assembly steps, which is consistent with an aggregation mechanism whose initial stages would involve a transition from isolated α-helix to CCs [22, 47].

Although originally raised against toxic oligomeric species of amyloid-β [48], A11 recognizes toxic oligomeric forms of amyloidogenic peptides of unrelated sequence, which makes it a valuable conformation-specific antibody [48]. Concomitant with the early CCs’ formation, SDS-sensitive oligomeric ApCPEB PLD species recognized by the A11 conformational antibody were formed during the first 72 h (Fig. 3d). The SDS-sensitive species evolved into SDS-resistant species, recognized by the OC conformational antibody, which binds a generic epitope common to diverse fibrillary amyloid and amyloid-like assemblies [49] (Fig. 3d). To confirm the amyloid-like nature of these OC-reactive assemblies, the fluorescence of Congo Red (CR) dye, whose intensity enhances when bound to amyloids [50, 51], was measured in aged samples (Fig. 3e). In agreement with CD data (Additional file 1: Figure S3a), QBP1 reduced the formation of ApCPEB PLD aggregates (Fig. 3e), which consisted of unbranched amyloid-like filaments of variable lengths and approximately 7–25 nm wide associated with annular assemblies, as visualized by transmission electron microscopy (TEM) (Fig. 3f).

The formation of short-lived A11-reactive species that evolved to a mature filamentous state with amyloid-like features was also found in Orb2A PLD [16]. In the light of these data, and to get insight into the nature of the transient A11-reactive, and mature OC-reactive ApCPEB PLD species, a conformational trapping assay was performed using two inhibitors: Amphotericin B (AmB) and (-)-epigallocatechin gallate (EGCG). As shown for other amyloids, such as Orb2 and Sup35 [16, 52], AmB and EGCG disturbed the assembly of ApCPEB PLD. In particular, it was trapped in an A11-reactive conformation by AmB and in an OC-reactive conformation by EGCG (Additional file 1: Figure S8). To further investigate the assemblies stabilized by these two compounds, the solvent-sensitive neutral dye Nile Red was used. Nile Red has a low quantum yield in polar environments, with a maximum emission at 655 nm. Its fluorescence intensity is unaffected by aromatic compounds such as AmB or EGCG but increases with a blue shift in nonpolar environments, exhibiting a maximum emission at 625 nm [53]. ApCPEB PLD multimers arrested by AmB showed intensities several folds higher than ApCPEB PLD alone or those aggregates arrested with EGCG (Fig. 3g). Thus, the oligomers trapped in the A11-reactive state exposed more hydrophobic residues to the solvent than the aggregates trapped by EGCG, which were more tightly packed. To investigate the toxicity of these diverse species, COS-7 cells were microinjected with each specific conformation. After microscopic visualization, A11-reactive species were found to induce cell death, contrary to OC-reactive species, or untreated ApCPEB PLD (Fig. 3h, i). Moreover, low concentrations of the A11 antibody (complex: A11, 100:1) neutralized the toxic phenotype of oligomers trapped in the A11-reactive state (Fig. 3h, i), supporting the notion that the exposure of hydrophobic surfaces in protein aggregates is associated with cell toxicity [54–56].

## Discussion

**Fig. 4:**
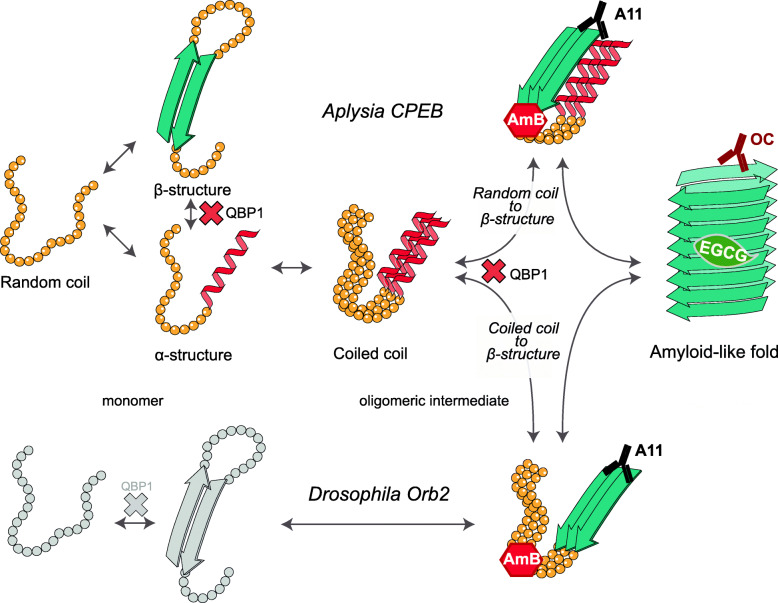
The possible role of CCs in the assembly pathway of ApCPEB PLD. Monomeric ApCPEB PLD fluctuates among β-, α-, and random coil conformations. In contrast to monomeric Orb2 PLD (bottom row), which remains as a mainly random coil in solution [16]. The association of α-helices present in monomeric ApCPEB PLD leads to the formation of CC species. CCs may mediate the conformational transition of neighboring random coil segments into β-sheet multimers [22, 47]. Alternatively, CCs may represent intermediate assembly structures facilitating their own conversion into β-sheet multimers. ApCPEB PLD and Orb2 PLD pathways converge in the formation of A11-reactive multimers, which can be trapped by AmB. A11-reactive multimers evolve to a common OC-reactive amyloid-like fold, which can be trapped by EGCG. QBP1, known to interact with poly-Q segments, blocks the monomer transition to a β-rich conformer which results in a reduction of M events in AFM-SMFS. In addition, QBP1 would potentially block the CCs transition to β-sheet multimers and hence, the amyloid-like assembly

The assembly pathway of the PLD from neuronal-specific CPEB isoforms in *Aplysia* and *Drosophila* converges at the oligomeric state. The presence of similar short-lived A11-reactive oligomers that evolve to OC- and CR-reactive amyloid-like filaments supports the idea that toxic A11 reactive oligomers are ephemeral species in functional aggregation [16, 52]. Yet, structural differences related to their divergent PLD sequences are found at the start of each assembly pathway. Both CPEB PLDs display a broad conformational polymorphism at the monomer level, as measured by AFM-SMFS, reinforcing the notion that monomers of amyloid-forming proteins fluctuate between a wide conformational space [57, 58]. Orb2 PLD exhibits small populations of α-helical conformers in solution at low pH, which significantly increases upon neutralization of His residues of the Q/H-rich segments [43]. By contrast, ApCPEB PLD is structured in solution and its early aggregation is mediated by the formation of CCs, as previously observed for other Q/N-rich proteins [22]. In this model, CCs might correspond to a stable oligomeric intermediate that would allow aggregation to be regulated [21, 47]. Once formed, CCs could mediate the conformational transition of neighboring random coil segments, or alternatively facilitate their own conversion into β-sheet multimers [22, 47] (Fig. 4). Solid-state NMR analysis on the aggregated full-length ApCPEB indicates that it has a rigid part associated with the PLD, and a dynamic part which corresponds to the RRM region [21]. This observation allowed the authors to propose a model for the gain of function during ApCPEB full-length aggregation. Here, the PLDs stack along the major axis constituting the filament core, while the dynamic RRM are exposed on the surface of the filament free to bind target mRNAs [21], in a similar manner than *Drosophila* Orb2 does [17]. Consistent with the data described here, which indicate that ApCPEB PLD forms helical CC structures at the beginning of its assembly pathway, the aforementioned NMR study revealed that the PLD not only is composed of β-sheet but also contains helical conformations. To gain a mechanistic understanding of the full-length ApCPEB aggregation mechanism, future studies should determine the atomic structure of the aggregated ApCPEB, as well as high-resolution structural insight on the conformational changes underlying monomer-to-aggregate transition.

Our findings show that divergent PLDs of neuronal-specific isoforms of CPEB family members from different species, in spite of sharing the formation of a common amyloid fold, have evolved to follow different assembly mechanisms. It has been reported that PLDs from different proteins are functionally interchangeable [16, 20], suggesting that attaining the amyloid-like fold may be sufficient to preserve the protein function. Although functional amyloids share biophysical properties with pathological ones [59], pathological amyloid aggregation seems to be a circumstantial, unregulated process. Contrary to this scenario, strict regulation is required in functional amyloidogenesis [60]. Recent studies on mammalian CPEBs, which possess a larger PLD with lower Q content compared to ApCPEB and Orb2, seem to feature a more sophisticated regulation of their amyloidogenesis [61–64]. These findings may suggest that the regulation of functional CPEB aggregation during the course of evolution, accompanied by diverse assembly mechanisms across species, became more finely controlled to satisfy novel needs as nervous systems became more complex.

## Conclusions

In conclusion, the assembly pathway of the prion-like domain of the neuronal-specific CPEB isoform in *Aplysia* is examined and compared with the previously reported for the prion-like domain of the neuronal-specific CPEB isoform in *Drosophila*. Our results show evidence for how divergent prion-like domain sequences assemble, following different mechanisms, into a generic amyloid-like fold, which is required to carry out a common function in the nervous system of diverse organisms.

## Methods

### Antibodies

The following antibodies were obtained from commercial sources: Rabbit polyclonal anti-Oligomer A11 (Thermo Fisher Scientific, Cat#AHB0052, RRID: AB_2536236), Rabbit polyclonal anti-Amyloid fibrils OC (Millipore Sigma, Cat#AB2286, RRID: AB_1977024), Goat anti-Rabbit HRP linked secondary antibody (Cell Signaling Technology, Cat# 7074, RRID: AB_2099233), Mouse monoclonal Anti-poly Histidine antibody (Sigma-Aldrich, Cat# H1029, RRID: AB_260015), and Horse anti-Mouse-IgG HRP linked (Cell Signaling Technology, Cat# 7076, RRID: AB_330924).

### Cloning

All constructs containing the N/Q-rich region of the neuronal-specific*Aplysia* CPEB (residues 1–160) were amplified by PCR using a plasmid containing the full-length ApCPEB (Addgene) as a template. The oligonucleotides used are listed in Additional file 1: Supplementary Table 2. All sequences were first subcloned into the pCR2.1 (Invitrogen) or pT7Blue (Novagen) vectors, using the *Escherichia coli* strains DH5α (Invitrogen) or XL1-Blue (Stratagene). After sequence verification, the fragment was inserted into (i) the pFS-2 vector for AFM-SMFS analysis, using the AgeI-SmaI restriction sites of the multi cloning site (MCS), which is localized in the I27 or Ubi carrier [34], or into (ii) the pET28a (+) vector (Novagen), using the NheI and XhoI restriction sites, to generate ApCPEB PLD and ApCPEB PLD-fusion proteins for all the remaining experiments, as described in [34]. All sequences were finally cloned in expression vectors.

### Protein expression

All isolated, carrier-guest monomers, and pFS-2 fusion proteins were expressed in the *E. coli* C41(DE3) or BL21(DE3) strains (Invitrogen). Bacterial cultures were grown at 37 °C until they reached an OD_595_ of 0.4–1.0. Then, their expression was induced by the addition of 1 mM IPTG for 4 h. Next, the bacterial pellets were lysed using lysozyme and sonication pulses as described in [16]. The recombinant proteins were purified by Ni^2+^-affinity and size-exclusion chromatography, using Histrap HP FPLC columns (GE Healthcare) and HiLoad 16/60 200 PG column (GE Healthcare), respectively, on an FPLC apparatus (ÄKTA Purifier, GE Healthcare). Protein concentration was determined by absorbance at 280 nm using the molar extinction coefficient of each protein.

### Computational analysis

Sequences were obtained from Uniprot and GenBank and analyzed in Pfam [65] and InterPro [66] to study the presence of conserved domains. Sequences were aligned using Clustal Omega with default parameters and represented using Boxshade. The pairwise identity as calculated by Clustal was represented as identity matrices. The N-terminal region was selected as the region from the N-terminus to the first RRM motif. RRM1 and RRM2 sequences were those identified by InterPro, while ZZ domains were identified by Pfam. The algorithms Coils [24], PairCoil2 [25], Marcoil [26], and PCOILS [27] were used for CC predictions. In Coils, a 28-residue window was used for the analysis. ZipperDB database was used to identify sequence segments with a tendency to form the steric zipper spines of amyloid filaments [67] (https://services.mbi.ucla.edu/zipperdb/). Disorder prediction was performed using a consensus artificial neural network prediction method, the Predictor of Naturally Disordered Regions PONDR-FIT (Molecular Kinetics). This meta predictor was developed by combining the outputs of several individual disorder predictors (PONDR-VLXT, PONDR-VSL2, PONDR-VL3, FoldIndex, IUPred, and TopIDP) [68].

### Nuclear magnetic resonance spectroscopy

2D ^1^H NOESY NMR spectra were obtained at 25 °C using samples at 1 mM in 10 mM KH_2_PO_4_, pH 4.7 with 10% D_2_O, by operating a Bruker AV 800 spectrometer (Bruker BioSpin) equipped with a ^1^H, ^13^C, ^15^N cryoprobe, and Z-gradients. The temperature was calibrated using an ethanol sample and the signal of the trimethyl moiety of DSS was used as an internal reference of the chemical shift. Selective pre-saturation or a WATERGATE module [69] were used to reduce the signal of the water present in the sample. The spectra were analyzed using TopSpin 2.0 (Bruker BioSpin). The assigned signals in the I27 spectra were based on previously reported data [70].

### Circular dichroism spectroscopy

Far-UV CD spectra of ApCPEB PLD samples, at a concentration of 2 μM in 10 mM KH_2_PO_4_ pH 4.7, were recorded using a JASCO-J810 spectropolarimeter (JASCO Inc.) equipped with a Peltier temperature control unit and using quartz cuvettes of 1-mmcell-path length. The secondary structure content was quantified by CD spectra deconvolution using the CDNN analysis program [71]. At the current state of the trained networks (CDNN version 2.0.3.188), the average error (%) for the prediction is 4.51%, 5.16%, and 6.20% for the 190–260 nm range using NNET_13, NNET_23, and NNET_33, respectively [71]. Once the first protein spectrum (hour 0) was recorded, the protein was incubated at 37.0 °C without stirring (with 0.02% NaN_3_), and subsequent spectra were collected over the following hours. For comparative purposes, and to minimize the impact of signal loss, the spectra recorded as a function of time were normalized by the signal of one of the typical minima used in the CC formation analysis. In particular, full trace intensities were divided by their own value acquired at 222 nm. The concentration of QBP1 and SCR (10 μM with a final DMSO concentration below 0.01%, to avoid DMSO interference with the ellipticity measurements) was 1:5 in molar excess. QBP1-M8 (Ac-WKWWPGIF-NH_2_) and SCR (Ac-WPIWKGWF-NH_2_) peptides with the N- and C-termini acetylated and amidated, respectively, were synthesized at the Proteomic Facility of the CBMSO/CSIC-UAM using solid-state Fmoc chemistry with N-terminal acetylation and C-terminal amidation.

### Association of 20-mer peptides from ApCPEB PLD

Four peptides derived from the residues 121–140 of the ApCPEB PLD were obtained from GenScript: “WQ” (WEQLQQQQLQLQQQLQQQLQH), “QW” (EQLQQQQLQLQQQLQQQLQHW), “DQ” (D*EQLQQQQLQLQQQLQQQLQH), and “DK” (D*EQLQQQQKQKQQQKQQQLQH), where D* is a dansyl moiety. Note that the first three peptides contain the Wt sequence whose Leu residues are naturally spaced to form a stabilizing hydrophobic interface between α-helices in a CC. The last peptide, DK, contains three Leu to Lys substitutions designed to disrupt that interface. The peptides’ purities were > 95% and their sequences were confirmed by mass spectroscopy, HPLC, and NMR spectroscopy. Their association at 5 °C in 10 mM KH_2_PO_4_ buffer was monitored by (i) 1D 1H NMR spectroscopy, using the sharp trimethyl signal of DSS as a concentration standard, and (ii) fluorescence spectroscopy, carried out in a Varioskan Flash microplate reader (Thermo Scientific), using an excitation wavelength of 280 nm to excite Trp and monitoring the dansyl emission band (530–550 nm).

### Isothermal titration calorimetry

ITC experiments were carried out in a VP-ITC microcalorimeter at 25 °C to examine the interaction of ApCPEB PLD with the minimal active core of the QBP1 (Ac-WKWWPGIF-NH_2_) and SCR (Ac-WPIWKGWF-NH_2_) peptides. The ApCPEB PLD protein samples were equilibrated in PBS pH 7.4 by size exclusion chromatography, and the equilibration buffer was used to prepare the peptide solutions. Protein/peptide binding was tested by successive injections of the protein (180 μM, 1 × 1 μL; 4–5 × 25 μl each) into the reaction cell loaded with peptide (495 μM QBP1 and 84 μM SCR) at a high final (peptide)/(protein) molar ratio. The apparent heat of reaction for each injection was obtained by integration of the peak area. The heat developed with the protein or peptide dilutions was determined in separate runs, loading either the sample cell (protein dilution) or the injection syringe (peptide dilution) with the buffer in the conditions used for the binding experiments. ApCPEB PLD (550 μM) titration into QBP1 (75 μM) was also carried out, but the complexity of the system and the lack of precise information on the distribution of the ApCPEB PLD conformations before and after complex formation precluded the quantitative analysis of the titration curves. The protein and ligand concentrations in the loading solutions were measured spectrophotometrically using their respective extinction coefficients.

### Congo Red binding assay

CR binding assays were performed using 10 μM ApCPEB PLD samples dialyzed in PBS, pH 7.0. Before the analysis, samples were aged for 10 days at 37.0 °C without stirring in the presence of 0.02% NaN_3_. After aging, the protein solutions were mixed with a 30 μM CR solution (in 5 mM sodium phosphate buffer + 300 mM NaCl, pH 7.5) and incubated at room temperature for 30 min. Both SCR and QBP1 peptides were used at a concentration of 50 μM. CR binding was calculated by measuring bathochromic and hyperchromic shifts in the samples, using a UV-visible spectrophotometer (Nanodrop, Thermo Scientific), and applying the following eq. CR (μmol/l) = A_540_/25,295 – A_480_/46,306 [72].

### Dot blot analysis

Immuno-dot blot analysis was performed using ApCPEB PLD aliquots at a concentration of 10 μM in PBS at pH 7.0. For EGCG and AmB studies, ApCPEB PLD in the monomeric state was prepared by denaturing it in 6 M Gdn·HCl, considered as the starting time of the reaction. Next, samples were diluted in 5 mM potassium phosphate + 150 mM NaCl, pH 7.4 to a final protein concentration of 2.5–5 μM. DMSO (AmB vehicle) or 4:1 M excess of AmB and EGCG was added. In all the cases, 2 μl of the sample was spotted onto a nitrocellulose membrane. After blocking the membrane for 1 h at room temperature with 10% non-fat milk in TBS containing 0.01% Tween-20, the membrane was incubated at room temperature for 1 h with the polyclonal specific anti-oligomer A11 antibody or the fibril-specific monoclonal antibody OC, diluted to 1:1000 in 3% BSA /TBS-T. The membranes were washed 3 times for 5 min each with TBS-T before incubating at room temperature for 1 h with the anti-Rabbit HRP linked secondary antibody diluted 1:5000 in 3% BSA/TBS-T. After washing the membranes 3 times in TBS-T buffer, the blots were developed with ECL Plus chemiluminescence kit from Amersham-Pharmacia (GE Healthcare).

### Transmission electron microscopy

Ten-micromolar ApCPEB PLD protein samples in PBS, pH 7.0, were used in TEM measurements. Five microliters of each sample were adsorbed onto carbon-coated 300-mesh copper grids (Ted Pella) and negatively stained for 30 s, using 1–2% uranyl acetate. Immediately before use, the carbon-coated grids were glow-discharged to enhance their hydrophilicity using an Emitech K100X apparatus (Quorum Technologies). Images were taken on a JEOL 1200EX II (Jeol Limited) electron microscope equipped with a CCD Megaview III camera (Olympus Soft Imaging) at an acceleration voltage of 80 kV.

### Single-molecule force spectroscopy based on atomic force microscopy

Double-blind SMFS measurements were performed using 2 μM of the pFS-2-ApCPEB PLD polyprotein in 10 mM Tris-HCl, pH 7.5, at room temperature. For comparative purposes, these conditions were the same as those used for the characterization of pathological aggregation-prone proteins [30], and for Orb2A PLD [16]. The selection of this buffer was based on the observation that low ionic strength usually disfavors aggregation and consequently, favors AFM-SMFS data acquisition.

Proteins were kept at 4 °C between sessions, and NTA-Ni^2+^ functionalized coverslips were used as a substrate to attach the pFS-2 polyproteins through the His-tag present at their N-terminus [34]. For experiments performed in the presence of peptides, the protein sample was incubated with QBP1 (20 μM in DMSO) and SCR (20 μM in DMSO), and the mixed samples were incubated overnight at 4 °C before performing the measurements. The custom-made single-molecule AFM used and its operation mode were described previously [73]. Before each experiment, the cantilever tip was cleaned for 5 min using a UV lamp (UV/Ozone ProCleaner™ Plus, Bioforce Nanosciences Inc.). The spring constant of each individual Si_3_N_4_ cantilever (MLCT-AUNM, Veeco Metrology Group; and Biolever, Olympus) was calculated using the equipartition theorem [74]. Experiments were performed at a constant pulling speed of 0.4 nm/ms in the length-clamp mode [75]. The data were analyzed using Igor Pro 6 (Wavemetrics) and the WLC model of polymer elasticity [37]:

$$ F(x)=\frac{k_BT}{p}\left[\frac{1}{4{\left(1-\raisebox{1ex}{$x$}\!\left/ \!\raisebox{-1ex}{${L}_C$}\right.\right)}^2}-\frac{1}{4}+\frac{x}{L_C}\right], $$

where *F* is the stretching force, *p* is the persistence length, *x* is the end-to-end length, and *L*_*c*_ is the contour length of the stretched protein. *L*_*c*_ and *p* are the adjustable parameters.

### Data analysis of AFM-based single-molecule force spectroscopy experiments

The criteria used for selecting bona fide single-molecule recordings were quite stringent and can be summarized as follows: (a) the inclusion of the N2B disordered polypeptide in the pFS-2 vector acts as a spacer to overcome the noisy proximal region of the force-extension spectra (50–70 nm if the polyprotein has been pulled from its termini [34]); (b) the recording should show several force peaks attributable to the unfolding of the Ubi repeats present in the pFS-2 vector as markers, whose *F*_*u*_ and Δ*L*_*c*_ values are described in the literature [35]; (c) the polyprotein should not show more force peaks than those expected from the design of the pFS-2 protein (excluding those derived from the unfolding of the ApCPEB due to its conformational polymorphism); (d) the total length of the unfolded molecule should not be larger than the total length of the extended polypeptide, taking into account the number of ubiquitin marker units unfolded and considering a gain in length of ≈ 0.38 nm per unfolded amino acid residue [76]; (e) since the ApCPEB is “force hidden” inside the carrier module of the pFS-2 vector, the force peak originated from the unfolding of the carrier module should always precede the force peaks corresponding to the unfolding of the grafted ApCPEB (Additional file 1: Figure S4 b-e); (f) any force peak that appeared at an extension shorter than that corresponding to the complete unfolding of the carrier module (≈ 26.6 nm for the carrier Ubi and ≈ 29.5 nm for the carrier I27) was excluded from our analyses as, in principle, it may have originated from spurious interactions between the nested ApCPEB and the carrier; and (g) only force data that showed a Δ*L*_*c*_ that, when summed, coincided exactly with the Δ*L*_*c*_ of ApCPEB (160 residues × 0.38 nm/residue ≈ 60.8 nm within a tolerance range of ± 4 nm) were included in our analyses (“b” and “c” in Additional file 1: Figure S4 b-e).

### Nile Red binding assay

Samples of ApCPEB PLD (5 μM in PBS, pH 7.0) in the presence or absence of 4:1 M excess of AmB and EGCG were removed at 100 min of incubation at 37 °C. A threefold excess of Nile Red was added, and the fluorescence spectrum was recorded using a Jobin Yvon Horiba FluoroMax 4 instrument (Horiba Jobin Yvon) and a 3-mm square quartz cuvette at 25.0 °C. Spectra were recorded in an emission wavelength range of 550–750 nm, with a 120 nm/min scan speed. Spectra of AmB and EGCG alone were also recorded and subtracted from the spectrum of the ApCPEB PLD complex. The excitation and emission wavelengths were calibrated using a fine Xe emission line and the Raman water peak, respectively.

### Single-cell protein microinjection

The COS-7 cell line was grown and maintained in supplemented DMEM with 10% (v/v) FBS. The day before performing the microinjection, cells were plated on a 35-mm dish at a density of 1 × 10^5^ cells/dish. The ApCPEB PLD proteins, at 2.5 μM in PBS 7.4, were incubated in the presence or absence of four-fold molar excess of AmB and EGCG for 100 min at 37.0 °C to allow complex formation. The ternary complex ApCPEB PLD:AmB:A11 was formed by incubation, for 3 h at room temperature, of the previously formed ApCPEB PLD:AmB with the A11 antibody (100:1, binary complex: A11). The samples were microinjected into the cytoplasm of single COS-7 cells (*n* = 100–200 cells per sample) along with fluorescein-labeled dextran (molecular weight 10000, Life Technologies) using a micromanipulator (Narishige). The experiments were carried out in a double-blind manner and were repeated three times per sample. Fluorescence micrographs were acquired using a CCD camera: model C4742-95-12ER (Hamamatsu Photonics). Ubi-MCS and VAMP2Cyt were microinjected as negative controls. 100% of cell viability was assigned by counting the number of fluorescein-positive cells 3 h after microinjection and using an IX70 fluorescence microscope (Olympus). During the three following days, the number of fluorescein-positive cells was counted every 24 h to calculate the cell survival rate. Data are represented as mean ± SEM. Two-way ANOVA and Bonferroni post-test were used as statistical methods for the analysis of the time-course survival curves. Statistical analyses were performed using GraphPad Prism 5 (GraphPad Software).

## Supplementary Information

**Additional file 1: Figures S1-S8** and **Supplementary tables 1 and 2**. **Figure S1**- [Sequence analysis of CPEB proteins]. **Figure S2** - [CC prediction of ApCPEB PLD]. **Figure S3** – [QBP1 interaction with ApCPEB PLD]. **Figure S4** - [Nanomechanical analysis of ApCPEB PLD using the “carrier-guest” strategy]. **Figure S5** - [Validation of the mechanical protection “carrier-guest” strategy used in AFM-SMFS experiments]. **Figure S6** - [Additional data from AFM-SMFS experiments]. **Figure S7** - [Association of 20-mer peptides from ApCPEB PLD]. **Figure S8** - [Different multimeric ApCPEB PLD species are trapped by AmB and EGCG]. **Supplementary Table 1** - [Summary of the AFM-SMFS analysis]. **Supplementary Table 2** - [Summary of the oligonucleotides used in this study].

## Data Availability

All data generated or analyzed during the current study are included in this published article, and its supplementary information files and underlying data are publicly available in figshare repository, at 10.6084/m9.figshare.c.5271908.v1.

## Abbreviations

AFM-SMFS: Single-molecule force spectroscopy based on atomic force microscopy
AmB: Amphotericin B
ApCPEB: *Aplysia californica* CPEB
CD: Circular dichroism spectroscopy
CC: Coiled coil
CR: Congo Red
CPEB: Cytoplasmic polyadenylation element-binding
Δ*L*_c_: Increase in contour length
DMSO: Dimethyl sulfoxide
DSS: sodium 2,2-dimethyl-2-silapentane-5-sulfonate
EGCG: (-)-Epigallocatechin gallate
FRET: Fluorescence resonance energy transfer
*F*_*u*_: Unfolding force
hCPEB3: Isoform 3 of human CPEB
hyCPEB4: Hydra CPEB
ITC: Isothermal titration calorimetry
M: Mechanically resistant
mCPEB3: Isoform 3 of mouse CPEB
MCS: Multicloning site
NM: Non-mechanically resistant
NMR: Nuclear magnetic resonance spectroscopy
NOESY: Nuclear Overhauser effect spectroscopy
pFS-2: Plasmid for force spectroscopy-2
PONDR-FIT: Predictor of naturally disordered regions
PLD: Prion-like domains
QBP1: Q-binding peptide 1
RRM: RNA-recognition motifs
SCR: Scrambled
SDS: Sodium dodecyl sulfate
TEM: Trasmission electron microscopy
Ubi: Ubiquitin
WLC: Worm-like chain
θ: Ellipticity
