# Supplementary material for: Divergent CPEB prion-like domains reveal different assembly mechanisms for a generic amyloid-like fold
Source: BMC Biol. 2021 Mar 11;19:43. doi: 10.1186/s12915-021-00967-9 (PMC7953810; doi:10.1186/s12915-021-00967-9)
Supplement: Supplementary file 1 — Additional file 1: Figures S1-S8 and Supplementary tables 1 and 2. Figure S1- [Sequence analysis of CPEB proteins]. Figure S2 - [CC prediction of ApCPEB PLD]. Figure S3 – [QBP1 interaction with ApCPEB PLD]. Figure S4 - [Nanomechanical analysis of ApCPEB PLD using the “carrier-guest” strategy]. Figure S5 - [Validation of the mechanical protection “carrier-guest” strategy used in AFM-SMFS experiments]. Figure S6 - [Additional data from AFM-SMFS experiments]. Figure S7 - [Association of 20-mer peptides from ApCPEB PLD]. Figure S8 - [Different multimeric ApCPEB PLD species are trapped by AmB and EGCG]. Supplementary Table 1 - [Summary of the AFM-SMFS analysis]. Supplementary Table 2 - [Summary of the oligonucleotides used in this study]. [file 12915_2021_967_MOESM1_ESM.docx]

**Divergent CPEB prion-like domains reveal different assembly mechanisms for a generic amyloid-like fold**

Rubén Hervás^1,2*#^, María del Carmen Fernández-Ramírez^1#^, Albert Galera-Prat^1^, Mari Suzuki^3,4^, Yoshitaka Nagai^3,5^, Marta Bruix^6^, Margarita Menéndez^6,7^, Douglas V. Laurents^6^ & Mariano Carrión-Vázquez^1*^

**Additional file 1**:

Figures S1-S8 and Supplementary tables 1 and 2. FigS1- [Sequence analysis of CPEB proteins]. FigS2 - [CC prediction of ApCPEB PLD]. FigS3 – [QBP1 interaction with ApCPEB PLD]. FigS4 - [Nanomechanical analysis of ApCPEB PLD using the “carrier-guest” strategy]. FigS5 - [Validation of the mechanical protection “carrier-guest” strategy used in AFM-SMFS experiments]. FigS6 - [Additional data from AFM-SMFS experiments]. FigS7 - [Association of 20-mer peptides from ApCPEB PLD]. FigS8 - [Different multimeric ApCPEB PLD species are trapped by AmB and EGCG]. Supplementary Table 1 - [Summary of the AFM-SMFS analysis]. Supplementary Table 2 - [Summary of the oligonucleotides used in this study]

**
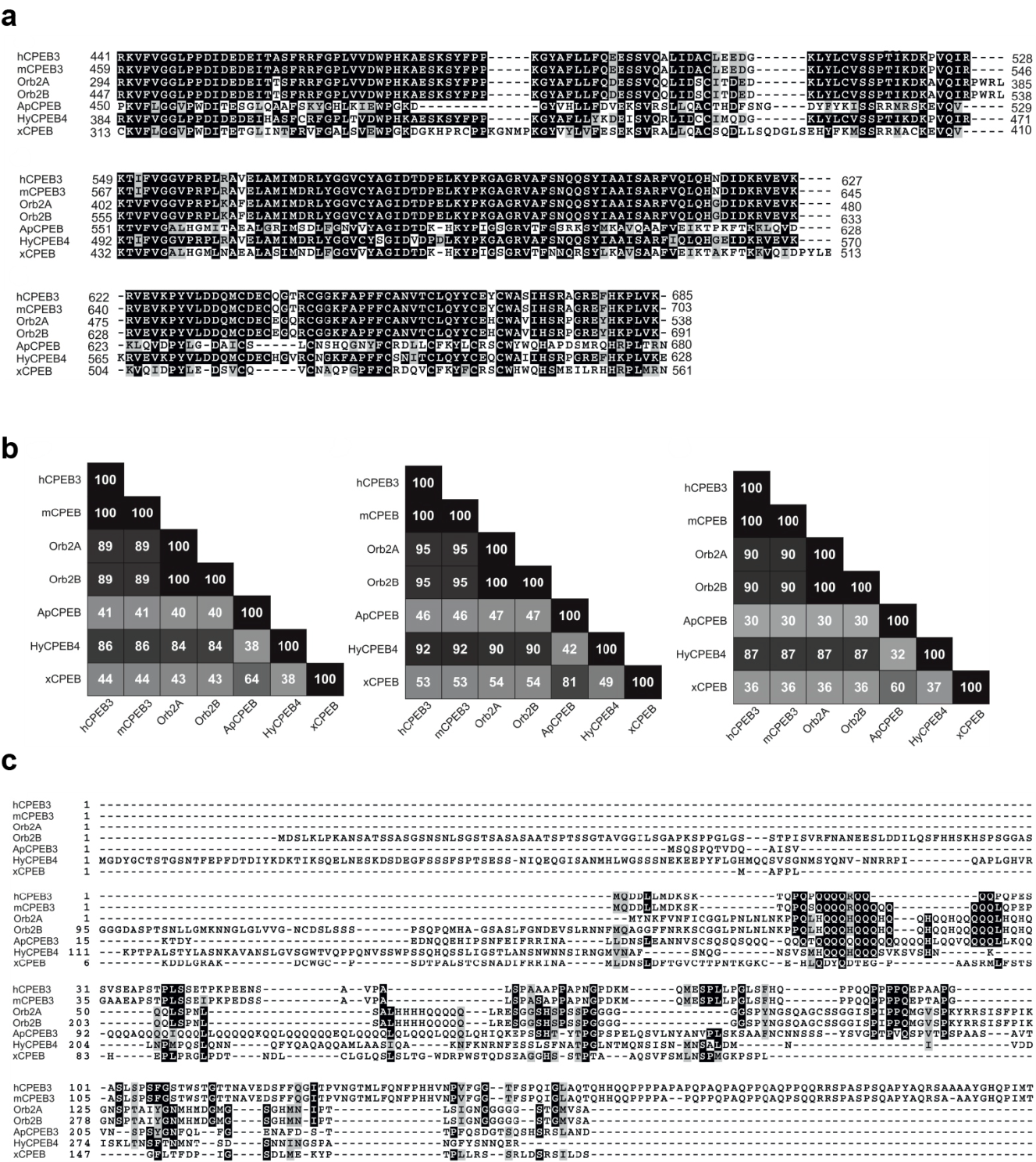
**

**Figure S1. Sequence analysis of CPEB proteins. a.** Sequence alignments of the structured C-terminal domains RRM1 (top panel), RRM2 (middle) and ZZ (bottom). **b.** Pairwise sequence identity represented as a matrix: RRM1 (left panel), RRM2 (middle) and ZZ (right). **c.** Sequence alignments of the N-terminal, disordered regions. The analyzed sequences correspond to the fragment from the N-terminus to the first residue of the RRM1 motif. Xenopus CPEB, xCPEB, is included as a family member lacking a PLD.


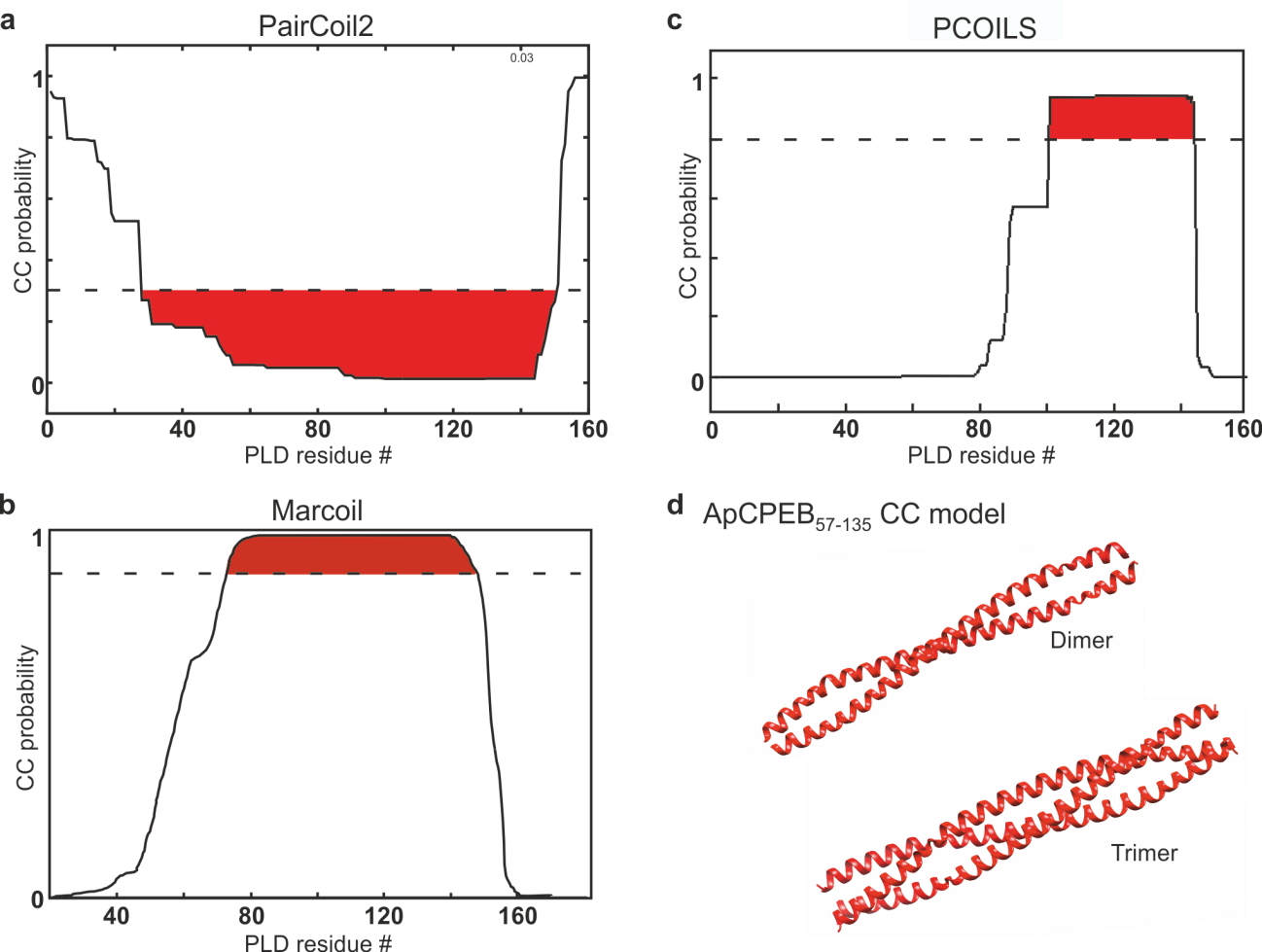


**Figure S2: CC prediction of ApCPEB PLD.** CC probability as calculated by PairCoil2 **a**), Marcoil **b**), and PCOILS **c**). Residues with the highest probability, with a threshold of 0-0.3 in a), 0.9-1 in b), and 0.8-1.0 in c), are highlighted in red. **d.** CC model for ApCPEB_57-135_, residues with the highest predicted score by Marcoil, as calculated by CCFold, an algorithm that produces CC models based on a statistical analysis of experimentally calculated structures [77].

**
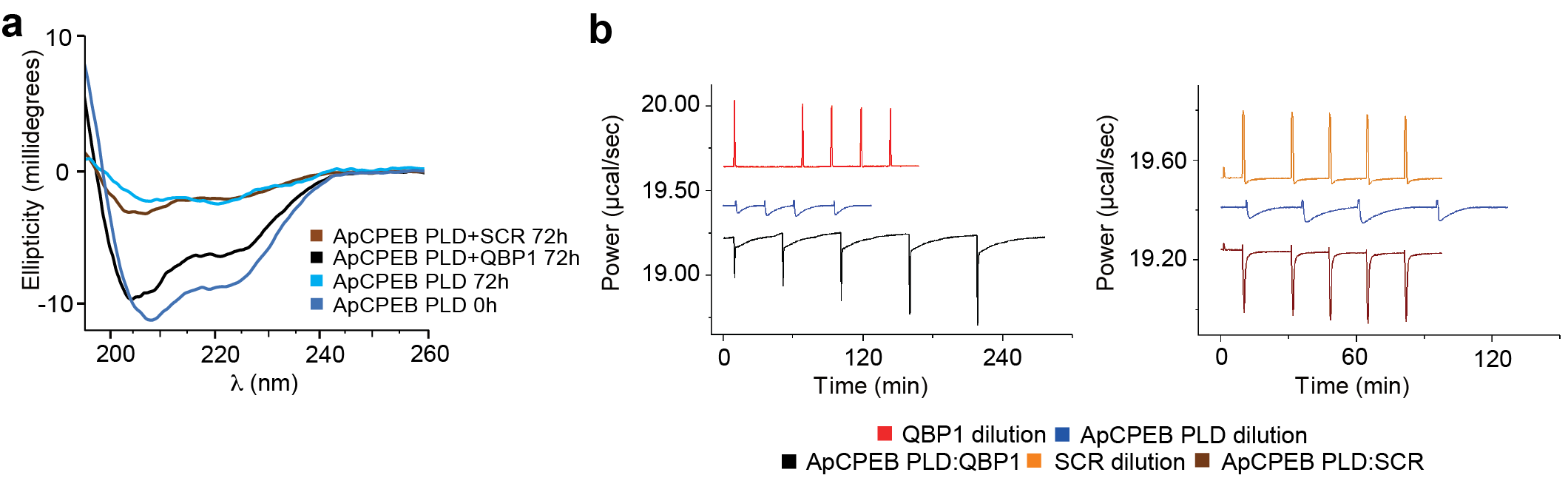
**

**Figure S3. QBP1 interaction with ApCPEB PLD. a.** Raw far-UV CD spectra of ApCPEB PLD. The far-UV ApCPEB PLD CD spectra show two minima at ~222 nm and 208 nm and a maximum below 200 nm, which are characteristic of α-helix-rich conformations. In the presence of QBP1, but not SCR peptide, the signal intensity over time is lowered. **b.** Representative calorimetric ITC traces for the injection of ApCPEB PLD into QBP1 (left panel) SCR (right panel) peptides. Traces in black and brown correspond to the heat released upon injection of ApCPEB PLD into the ITC cell loaded with QBP1 or SRC, respectively. Blue, red, and orange traces correspond to the ApCPEB PLD, QBP1, and SRC dilutions, respectively.

### **
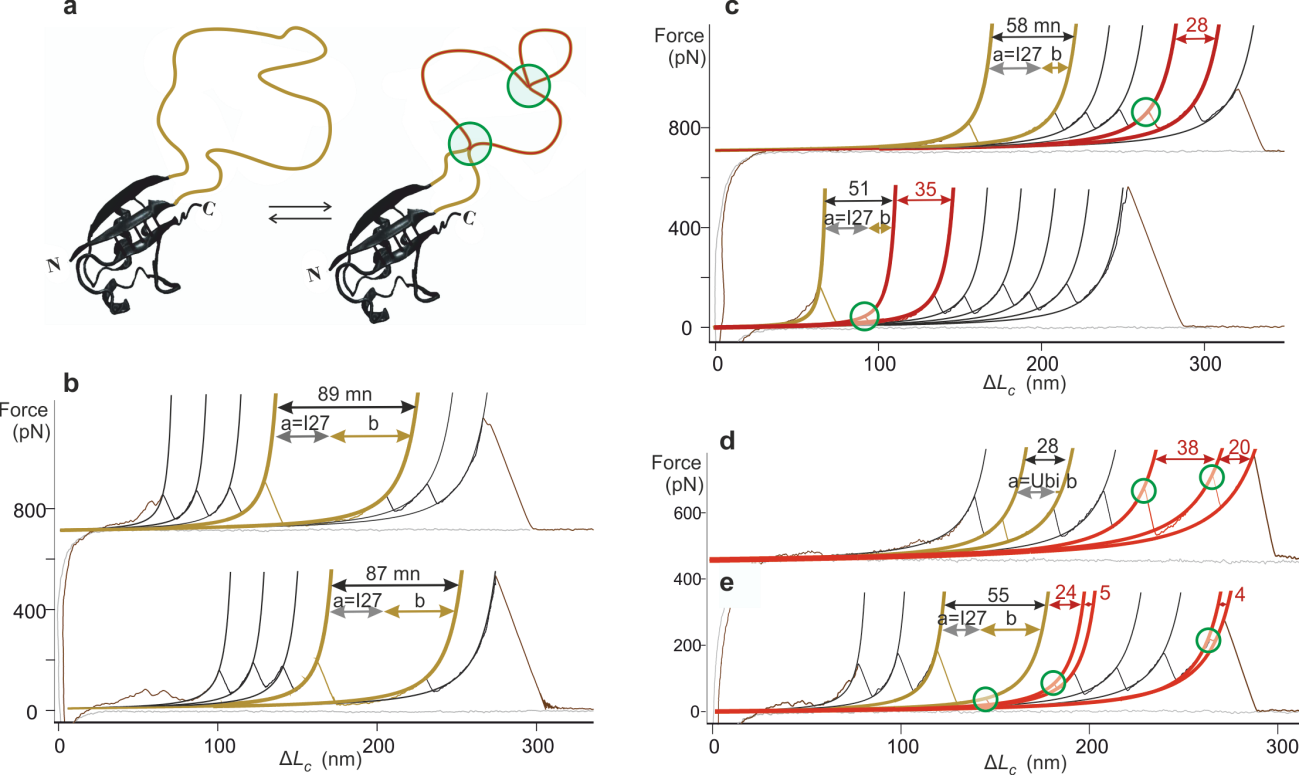
**

### **Figure S4**. **Nanomechanical analysis of ApCPEB PLD using the “carrier-guest” strategy.** **a**. Schematic representation of the carrier-guest strategy used in the pFS-2-Ubi-ApCPEB PLD polyprotein, in which Ubi is used as a carrier (black, PDB accession code: 1UBQ). A schematic of an NM and M conformation adopted by ApCPEB PLD are represented in yellow and red, respectively. The intramolecular interactions that would originate resistance barriers (i.e., force peaks) in the AFM-SMFS recordings are represented by green circles. **b-e**. Representative force-extension recordings of pFS-2-Carrier (I27/Ubi)-ApCPEB PLD. The elasticity of the stretched proteins was analyzed fitting the recordings to the WLC model of polymer elasticity [78]. The mechanical protection approach reveals different conformations adopted by the ApCPEB PLD, from NM (in yellow, **b**) to M (in red, **c-e**) conformations. M conformations show variability in *F_u_* and Δ*L_c_* values, which reflects the conformational diversity acquired by the PLD at the monomer level. The carrier module (gray arrow) must unfold completely (“a” represents the Δ*L_c_* upon unfolding) before the force can access the ApCPEB PLD guest: “b” and “c” represent the Δ*L_c_* for NM and M regions of ApCPEB PLD, respectively. The sum of “b” + “c” corresponds to the complete length of the stretched ApCPEB PLD, which is ≈ 60.8 nm, calculated as: 160 residues x 0.38 nm/residue [76]. The complete extension of the carrier I27 is ≈ 29.5 nm, while for Ubi is ≈ 26.6 nm. Thus, the sum of “a”+”b”+”c” must equal the theoretical value of ≈ 90.3 nm (using I27 as carrier), or ≈ 87.4 nm (using Ubi as carrier), within the experimental uncertainty of ≈ ± 4 nm.

**
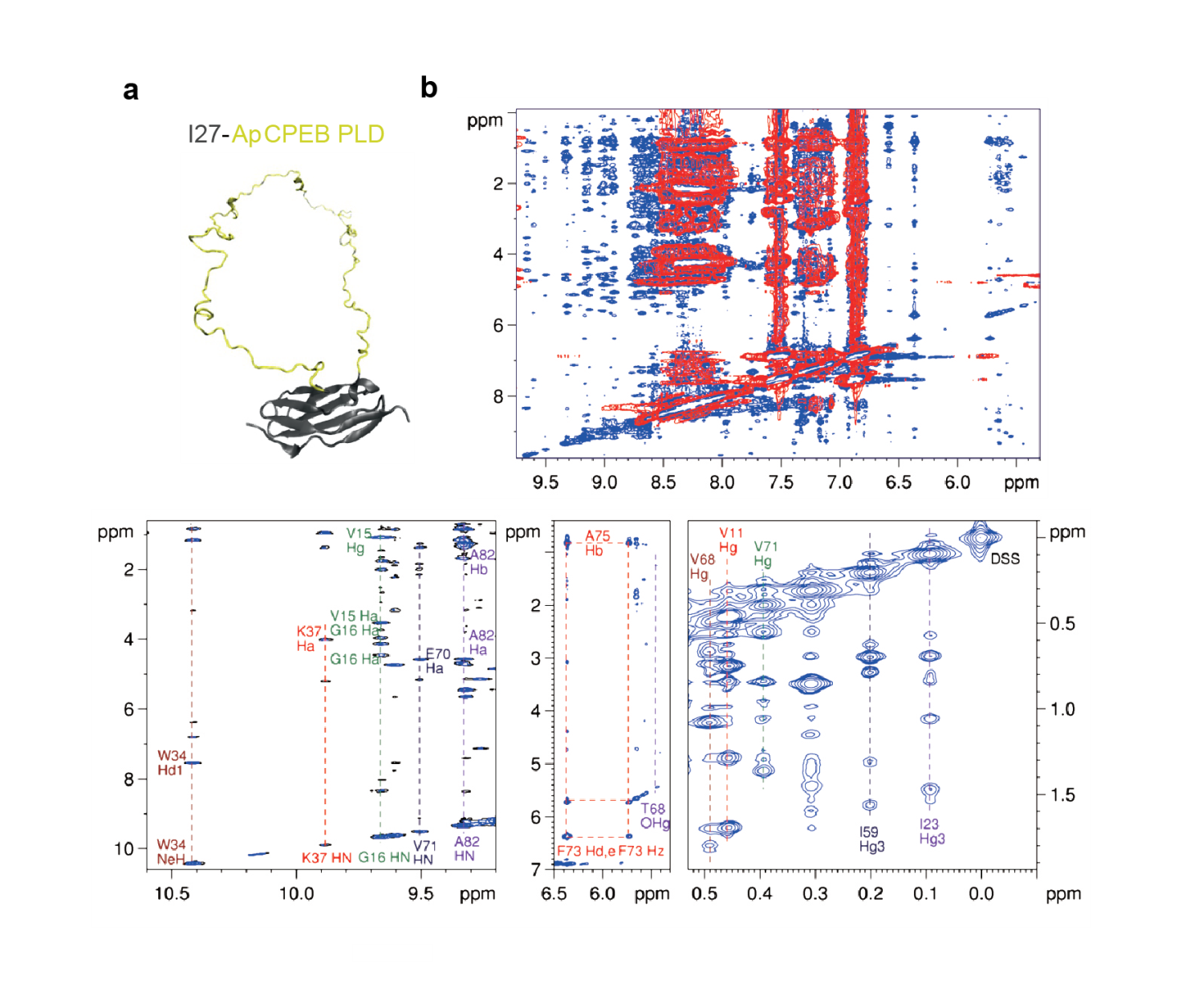
**

**Figure S5. Validation of the mechanical protection “carrier-guest” strategy used in AFM-SMFS experiments**. **a.** Schematic cartoon representation of I27-ApCPEB PLD carrier-guest construction colored in gray and yellow, respectively. The representation was prepared with MODELLER and displayed by Visual Molecular Dynamics v1.8.6, using atomic coordinates for titin I27 (PDB accession code: 1TIT) and off-template modeling for ApCPEB PLD. **b**. 2D ^1^H NOESY spectra of ApCPEB PLD (red) and I27-ApCPEB PLD (blue). The upper panel shows the downfield region of the spectra. Whereas I27-ApCPEB PLD gives rise to many signals with a diverse range of chemical shifts, those of ApCPEB PLD are clumped together in regions whose chemical shift is typical of disordered peptides and proteins [79]. Additional 3D heteronuclear spectra would be necessary to detect small content of β- or α-structures. The bottom panels show selected local regions of the fusion protein spectrum and some peaks which are unambiguously assigned to ^1^H in I27 labeled, which is conclusive evidence that the native structure of I27 is conserved.

**
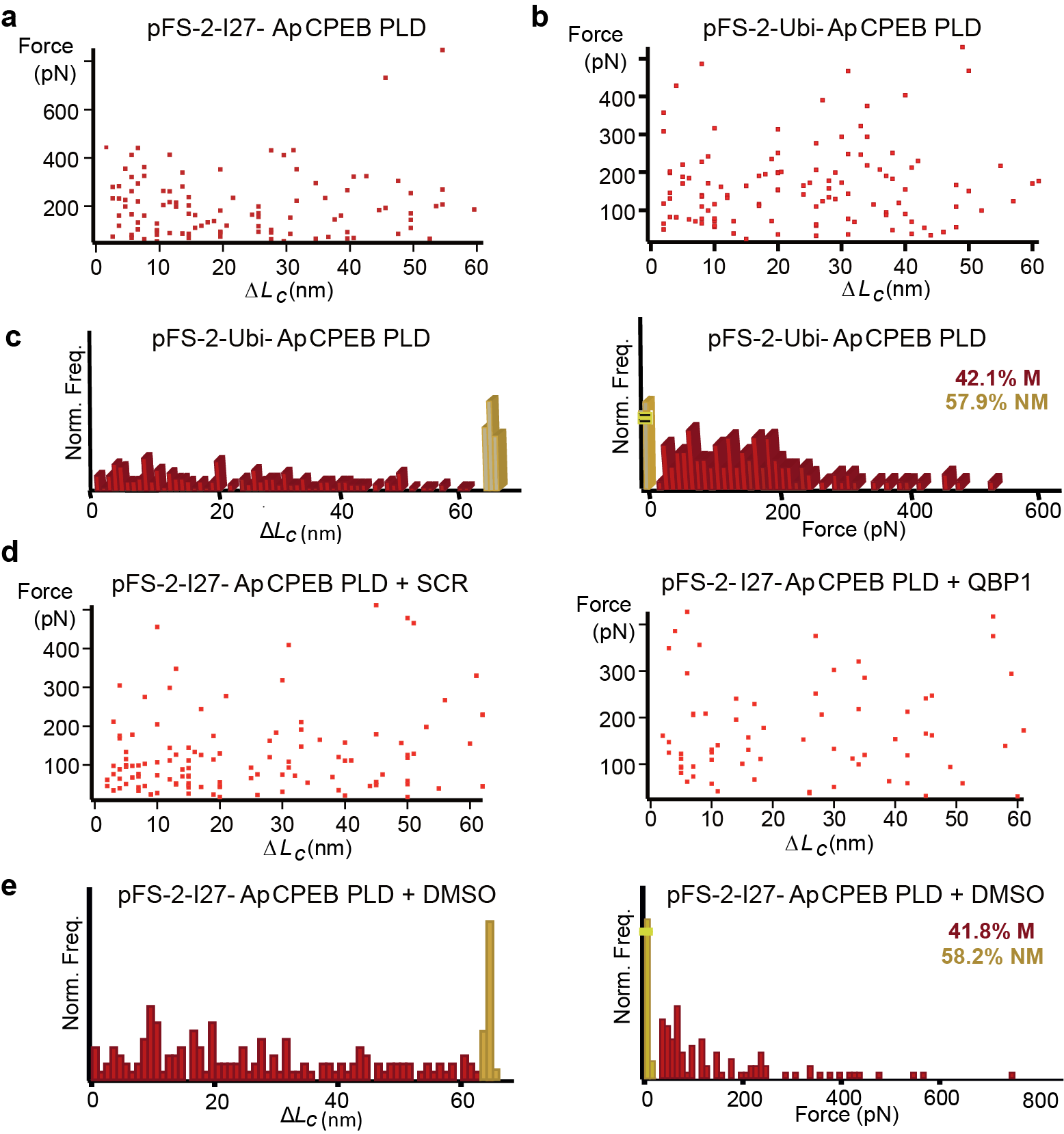
**

**Figure S6. Additional data from AFM-SMFS experiments. a.** *F*_u_ and Δ*L_c_* scatter plots for the M conformers from pFS-2-I27-ApCPEB PLD. The uncorrelation between *F*_u_ and Δ*L_c_* suggests the lack of formation of preferred structures. **b.** *F*_u_ and Δ*L_c_* scatter plots for the M conformers from pFS-2-Ubi-ApCPEB PLD. **c.** Δ*L_c_* (left panel) and *F*_u_ (right panel) histograms from pFS-2-ApCPEB PLD using Ubi as a carrier (n = 155). **d.** *F*_u_ and Δ*L_c_* scatter plots for the M conformers from pFS-2-I27-ApCPEB PLD + SCR (left) and pFS-2-I27-ApCPEB PLD + QBP1 (right) **e.** Δ*L_c_* (left panel) and *F*_u_ (right panel) histograms from pFS-2-I27-ApCPEB PLD measurements performed in the presence of 0.01 % DMSO. This DMSO concentration, used to dissolve the QBP1 and SCR peptides, was shown not to perturb the Δ*L_c_* and *F*_u_ distributions (n = 97).

**
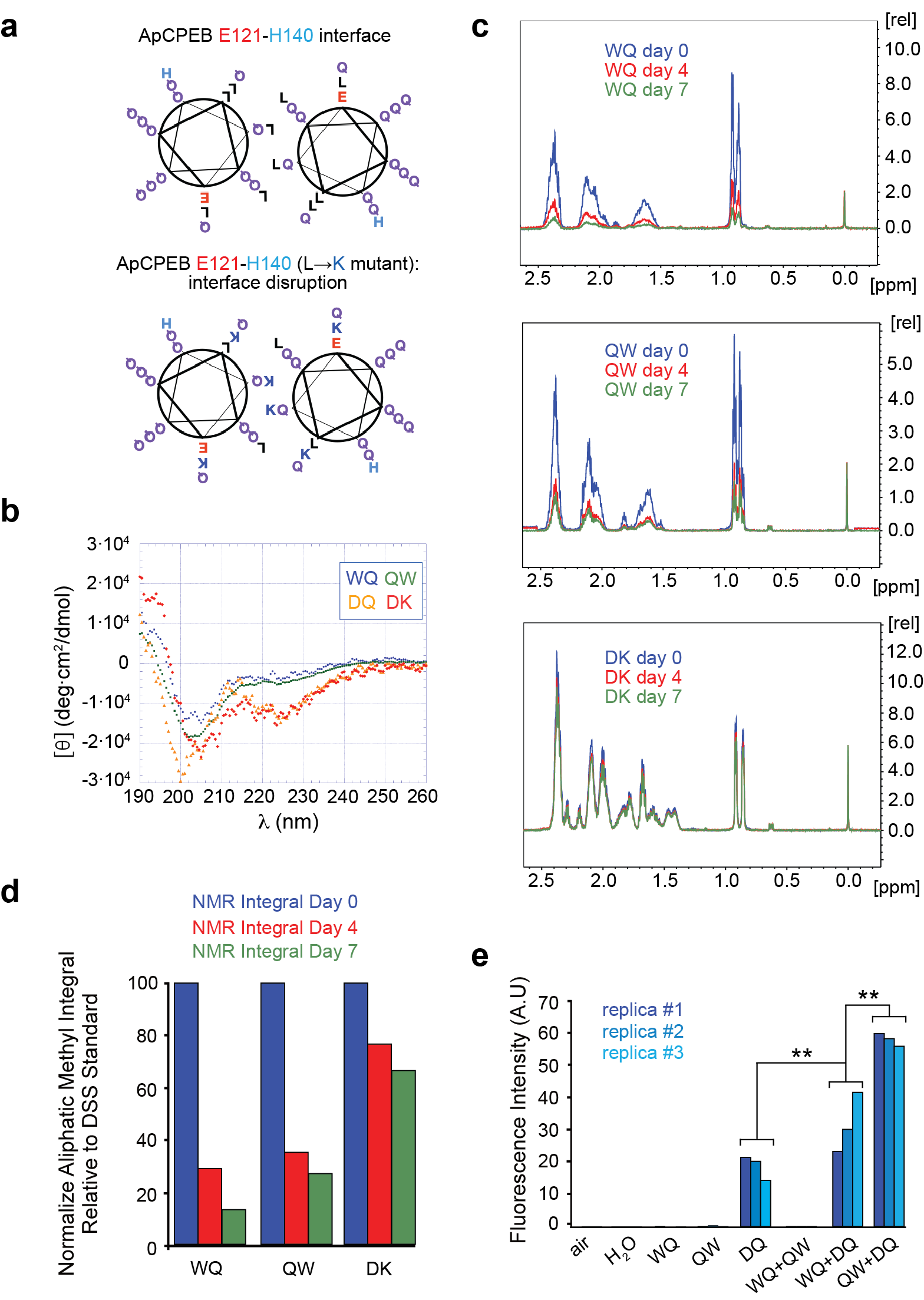
**

**Fig. S7: Association of 20-mer peptides from ApCPEB PLD. a.** Experimental design. Helical wheel diagram of two coiled helices of Wt and mutant ApCPEB heptads. The leucine residues could be positioned on the same side of an α-helix and therefore could form a stabilizing hydrophobic interface in a CC. This nonpolar surface would be disrupted in the “DK” variant, which contains three Leu to Lys substitutions producing charge/charge repulsion. **b**. The CD spectra of the four peptides, recorded at 5ºC in 10 mM KH_2_PO_4_ buffer with a scan speed of 20 nm/min and averaging 8 scans per spectrum and peptide concentrations of 13, 38, 59 and 53 µM for WQ, QW, DQ and DK, respectively, are shown below. They reveal a mixture of helical and statistical coil conformers and strongly suggest that the “DK” peptide is at least as helical as the WQ and QW peptides. **c**. 1D ^1^H spectra of the peptides WQ, QW and DK after 0 (blue), 4 (red) and 7 (green) days of incubation. The peak heights of the individual spectra have been adjusted so that the peak height of the DSS standard (at 0.00 ppm) is constant. The peaks belonging to the peptides, such as the aliphatic methyl signals resonating between 1.00 and 0.80 ppm, decrease sharply for WQ and QW and modestly for DK. **d**. NMR integral (from panel **c**) as a function of time. The aliphatic methyl peak integrals of WQ (left columns), QW (middle columns) and DK (right columns) relative to the DSS after 0 (blue), 4 (red) and 7 (green) days of incubation. The peptide carrying the 3 Leu to Lys substitutions aggregates less, as was anticipated from the expected disruption of the coiled-coil hydrophobic interface. **e**. FRET efficiencies for 20-mer peptides from ApCPEB PLD. FRET efficiencies when the W donor was located at the C-terminus of the peptide were significantly higher than when it was located at the N-terminus, suggesting the anti-parallel CC configuration as the most stable one. Data are represented as three independent experimental replicates. **p<0.01 (One-way ANOVA and Tukey post-test).


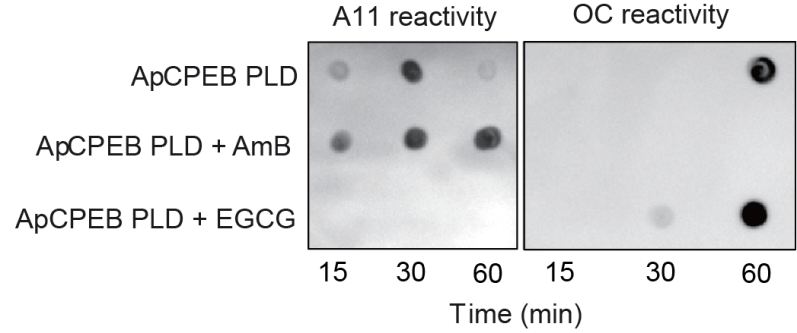


**Figure S8. Different multimeric ApCPEB PLD species are trapped by AmB and EGCG**. Immuno-dot blot analysis of the ApCPEB PLD multimers trapped by AmB or EGCG. Species trapped by EGCG interact with the OC antibody, whereas species trapped by AmB react with the A11, but no with the OC antibody.

| **Protein Construct** | **n** | **M (%)** | **NM (%)** | **#peaks/mol.** | ***F vs*. Δ*L_c_*** |
| --- | --- | --- | --- | --- | --- |
| **Ubi-ApCPEB PLD** | 155 | 42.1 | 57.9 | 1.87 | Uncorrelated |
| **I27-ApCPEB PLD** | 145 | 40.0 | 60.0 | 1.72 | Uncorrelated |
| **I27-ApCPEB PLD+QBP1** | 186 | 21.5 | 78.5 | 1.68 | Uncorrelated |
| **I27-ApCPEB PLD+SCR** | 144 | 43.3 | 56.7 | 1.77 | Uncorrelated |
| **I27-ApCPEB PLD+DMSO** | 97 | 41.8 | 58.2 | 1.81 | Uncorrelated |

**Supplementary Table 1. Summary of the AFM-SMFS analysis**. # peaks/molecule refers to the average number of M regions (force peaks) per molecule. The values reported are calculated considering only M conformers, excluding those monomers exhibiting an NM conformation (which results in 0 force peaks).

| **Construct** | **Oligonucleotide (sequence 5’ to 3’)** |
| --- | --- |
| ApCPEB PLD 5’ | CTA*GCTAG*CCATGCAAGCCATGGCCGT |
| ApCPEB PLD 3’ | CCG*CTCGAG*CTACTATGGAACCAGGCGTGTA |
| I27-ApCPEB PLD 5’  Ubi-ApCPEB PLD 5’  pFS-2-I27-ApCPEB PLD 5’  pFS-2-Ubi-ApCPEB PLD 5’ | CCAA*ACCGGT*ATGCAAGCCATGGCCGT |
| pFS-2-I27-ApCPEB PLD 3’  pFS-2-Ubi-ApCPEB PLD 3’ | TCC*CCCGGG*TGGACCAGGCGTGTA |

**Supplementary Table 2. Summary of the oligonucleotides used in this study.** The restriction sites introduced by PCR into the amplified sequences are highlighted in italics. Underlined sequences correspond to stop codons. The extra sequences added to the end of each restriction site were chosen on the basis of the recommendations from New England Biolabs, which enhance the digestion efficiency of linear DNA sequences. All oligonucleotides used were purchased from Sigma-Aldrich.

**References**

77. Guzenko D, Strelkov S V. CCFold: Rapid and accurate prediction of coiled-coil structures and application to modelling intermediate filaments. Bioinformatics. 2018; 34:215-222.

78. Bustamante C, Marko JF, Siggia ED, Smith S. Entropic elasticity of λ-phage DNA. Science. 1994; 265:1599-600.

79. Lopez-Alonso JP, Bruix M, Font J, Ribo M, Vilanova M, Jimenez MA, et al. NMR spectroscopy reveals that RNase A is chiefly denatured in 40% acetic acid: implications for oligomer formation by 3D domain swapping. J Am Chem Soc. 2010;132:1621–30.
